# Implementation of Integrative Nursing for Patients With Cancer Receiving Inpatient Care: Protocol for a Convergent Parallel Mixed Methods Evaluation

**DOI:** 10.2196/74405

**Published:** 2025-10-21

**Authors:** Lea Raiber, Beate Stock-Schröer, Johanna Thiele, Klaus Kramer

**Affiliations:** 1 Section Integrative Medicine Department of General and Visceral Surgery Ulm University Medical Center Ulm Germany; 2 Interprofessional Graduate School Integrative Medicine and Health Health Department Witten/Herdecke University Witten Germany

**Keywords:** complementary nursing intervention, inpatient care, integrative medicine, integrative oncology, nursing counseling, quality of care, study protocol, supportive care, symptom management

## Abstract

**Background:**

Integrative nursing (IN) involves the application of external naturopathic nursing interventions, such as compresses, embrocations, and therapeutic baths and washes. As part of a university hospital project, patients receiving oncology care in participating wards receive IN interventions as supportive care during their hospital stay as part of a consultation service.

**Objective:**

This study aims to investigate the acceptance, feasibility, and contextual conditions of implementing IN in inpatient care and to evaluate perceptions, experiences, and perceived impact of IN interventions from multiple stakeholder perspectives.

**Methods:**

We used a convergent parallel mixed methods approach guided by the Consolidated Framework for Implementation Research. The evaluation consists of 5 substudies reflecting multiple perspectives on the project. Patients, relatives, and hospital staff will participate. Substudies include a single-arm pre-post questionnaire (substudy 1) and semistructured interviews (substudy 2) with patients, a cross-sectional survey of relatives (substudy 3), semistructured interviews with health care professionals (substudy 4), and analysis of project-related documentation (substudy 5). Qualitative data will be analyzed using qualitative content analysis, and quantitative data will be analyzed using descriptive and inferential statistical methods.

**Results:**

Following separate analyses of each substudy, the findings will be integrated and triangulated to generate overarching meta-inferences. The recruitment phase lasted from October 2023 to January 2025. Data collection was completed in March 2025. As of October 2025, after data verification and plausibility checks, data analysis is ongoing. The first results are expected to be published in 2026.

**Conclusions:**

This study presents a mixed methods research protocol aimed at exploring the implementation of IN within a university hospital setting. It is expected to provide a theory-based contribution to IN implementation in inpatient care while also offering insights into its potential effects at the patient level. The study is anticipated to advance understanding of how IN can be sustainably embedded in hospital practice and to provide actionable insights for improving patient-centered supportive care.

**Trial Registration:**

German Clinical Trials Register DRKS00032318; https://drks.de/search/de/trial/DRKS00032318

**International Registered Report Identifier (IRRID):**

DERR1-10.2196/74405

## Introduction

### Background

In the following, we differentiate among complementary and integrative medicine (CIM), integrative nursing (IN), and integrative oncology (IO). CIM broadly refers to approaches that combine conventional medicine with complementary therapies [1,2]. IN denotes a nursing-specific framework emphasizing complementary, nonpharmacological, nurse-delivered interventions embedded within conventional care [3]. IO describes the broader integration of such approaches into oncology practice, encompassing medical, nursing, and psychosocial perspectives [4].

Patients with cancer show a significant demand for CIM, which involves both traditional and complementary approaches [5,6]. The global prevalence of CIM use among patients with cancer ranges widely from 16.5% to 93.4%, with an average prevalence of 41.1% reported for Germany [5]. This trend is further supported by findings from a survey conducted at German university hospitals, in which nearly half of the patients surveyed (48%) reported previous or current use of CIM in relation to their disease [7].

IN refers to a care approach that combines conventional nursing practices with evidence-informed complementary methods, aiming to holistically address the physical, emotional, social, and spiritual needs of patients [8-10]. IN interventions include a range of naturopathic, nonpharmacological nursing interventions, such as compresses, embrocations, and therapeutic baths and washes [10-12]. In an oncology setting, these IN interventions aim to prevent and manage symptoms and side effects associated with conventional treatment [10,13]. In recent years, a growing body of research, including reviews and evidence syntheses, has emerged on IN interventions both in general [14-16] and in relation to specific symptoms such as mucositis [17], pain [18], and chemotherapy-induced peripheral neuropathy [19]. In addition, individual studies have investigated specific symptom burdens, such as fatigue [20,21] or sleep quality [22,23], as well as postoperative support during the hospital stay [24,25]. A mixed methods systematic review taking a broader perspective on external applications suggests potential benefits for managing cancer-related symptoms but also identifies limitations, including low study quality and methodological inconsistencies among the included studies [15]. A similar pattern is reflected in an overview of systematic reviews, which indicates a generally positive influence of CIM on patient-reported outcomes in patients with cancer, for example, acupuncture has been associated with pain relief, while also acknowledging methodological limitations and, in part, inconclusive findings [26].

IN therefore plays a relevant role in the field of CIM in oncology [10,27,28], with IN interventions being implemented as part of IO programs [27,29]. Ben-Arye et al [30] highlighted the positive impact of nurse involvement in guiding patients to self-administer IO treatments. However, in this context, the influence of IN interventions at the patient level (eg, patient-reported outcomes) and the institutional level (eg, structural factors) have been examined only as part of multimodal programs [31-33].

In addition, research projects in Germany have focused on implementing IN interventions in health care, particularly in outpatient settings [34-36] and in pediatric inpatient care [37-40]. Recent studies in these contexts have demonstrated the considerable potential of IN interventions to support patients with cancer in managing cancer-related symptoms [41], improving patient activation [42], and enhancing their quality of life [35]. However, these findings are only partially generalizable to adult inpatient populations. Consequently, research on the feasibility, implementation, and evaluation of IN for patients with cancer in inpatient settings remains limited in Germany. Despite the considerable growth of research on CIM and IO in recent years, the specific role of IN is still underrepresented in the literature. Evidence on the systematic implementation and sustainability of IN interventions in inpatient oncology settings remains scarce.

In this context, the IMPLEMENT-UKU (Implementation of Integrative Nursing at the Ulm University Hospital) project offers an IN consultation service for patients on participating wards. The project provides a supportive, complementary care approach that uses external naturopathic nursing interventions to supplement conventional treatments in a patient-centered manner.

### Purpose of This Study

This evaluation study is embedded within the IMPLEMENT-UKU project and will examine the implementation of IN through a comprehensive, accompanying scientific investigation that integrates the perspectives of patients, their relatives, and hospital staff. It aims to address the following research questions: (1) To what extent can IN be implemented as intended on the participating wards, including the delivery of IN (eg, number of IN consultations, IN interventions) and its acceptance and perception among patients, relatives, and health care professionals (eg, evaluation of IN, perceptions of IN interventions)? (2) What are the key contextual factors influencing the implementation of IN, including identification of relevant contextual factors and assessment of the implementation process, such as barriers and facilitators? (3) What are the indicators of potential effects of IN at the patient level, with a focus on patient-reported outcomes (eg, well-being, quality of life, self-efficacy, and symptom burden)?

This study aligns with the research priorities outlined in the “German Research Agenda for Nursing Oncology,” particularly regarding the management of disease-related symptoms and treatment side effects, as well as the promotion of quality of life across different phases of health and disease [43].

## Methods

### Project IMPLEMENT-UKU: Setting and Intervention

**Figure 1:**
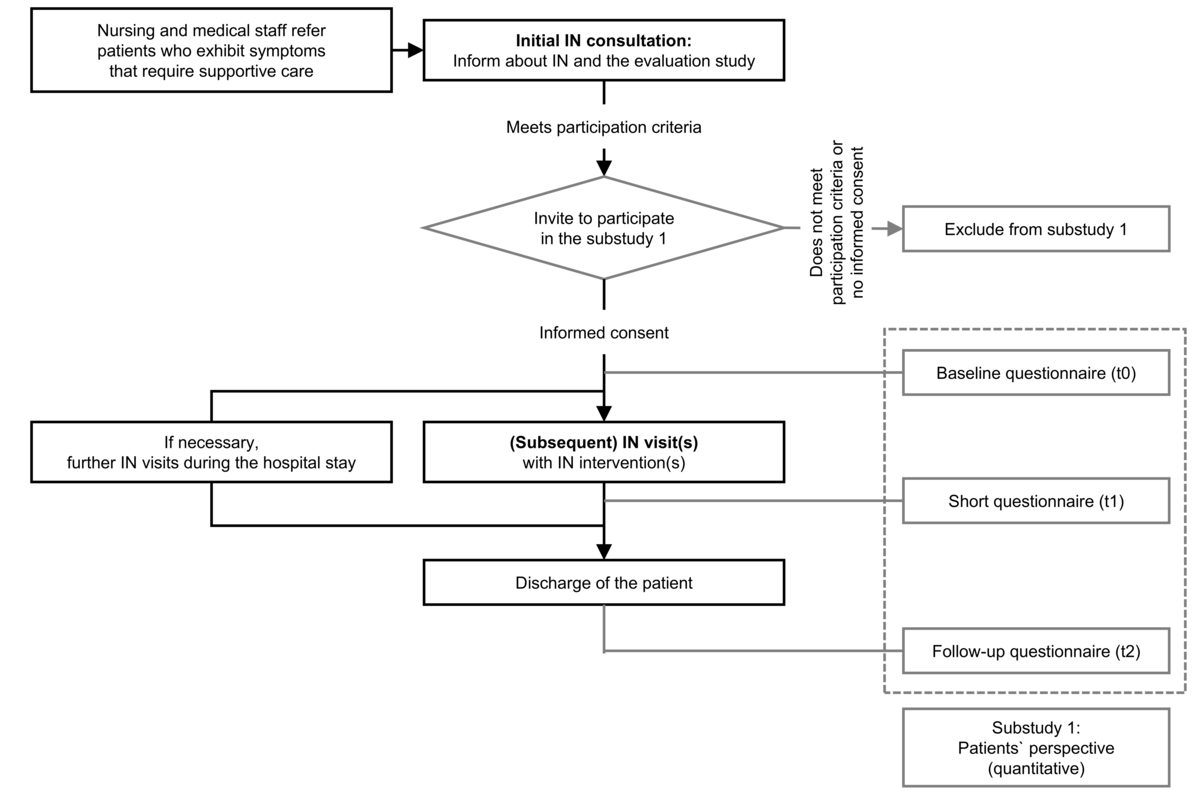
Flowchart of the patient care pathway within the IMPLEMENT-UKU (Implementation of Integrative Nursing at the Ulm University Hospital) project and in substudy 1.

The IMPLEMENT-UKU project and its accompanying evaluation study are being carried out as a single-center initiative at the University Hospital Ulm. Within the framework of the project, patients with cancer on participating wards receive IN interventions as supportive care during their hospital stay as part of a consultation service. Figure 1 illustrates a flowchart of the patient care pathway within the project.

Patients will be referred to an initial IN consultation by nursing or medical staff on the participating wards. Referrals will be based on pertinent and acutely distressing symptoms and problems, such as restlessness, pain, or cancer-related side effects, which are relevant to care and indicate the need for supportive interventions. Following referral, patients will receive an initial IN consultation, which may be followed by additional IN visits and interventions throughout their hospital stay. During the initial IN consultation, the integrative nurse will provide information about IN, the overarching IMPLEMENT-UKU project, and the ongoing evaluation study. The first IN intervention will be conducted directly after this consultation. The number of subsequent IN visits and interventions may vary depending on patient needs and the duration of hospitalization.

Depending on each patient’s symptom burden and individual preferences, patient-tailored IN interventions will be offered alongside routine care. To ensure consistent application, these interventions will be guided by a symptom-driven IN intervention catalog (Table 1). The IN catalog was developed through an expert consensus process, drawing on external naturopathic nursing textbooks [11,12,44-47], academic literature [14,15,18,19,48], and recommendations from previous projects and guidelines [36,49-51]. To ensure intervention fidelity, standard operating procedures are available for all IN interventions.

IN interventions are carried out by nurses who are specially trained in IN. These nurses have completed 3 years of vocational nursing education and have extensive professional experience. In addition, they have received training in CIM and hold qualifications in external naturopathic interventions, such as advanced training in compresses and wraps (40 teaching hours), rhythmic embrocation according to Wegman and Hauschka (80 teaching hours), and mindful body care and therapeutic washing (16 teaching hours), provided by the Association for Anthroposophical Nursing in Germany.

Although IN interventions are tailored to individual patient needs, fidelity is ensured through detailed standard operating procedures, structured nursing training, and standardized project-related documentation by nursing staff (substudy 5). This approach enables monitoring of adherence to the intervention catalog and allows systematic examination of variability, including its relationship to patient characteristics and contextual influences.

**Table 1:** Catalog of integrative nursing (IN) interventions in the IMPLEMENT-UKU (Implementation of Integrative Nursing at Ulm University Hospital) project (as of August 2023).

Symptoms	IN interventions^a^
Appetite loss	Liver compress with yarrow tea or oil^b^
Respiratory insufficiency and respiratory infections	Sternum compress with thyme oil^b^, plantago bronchial balsam^c^ Embrocation with thyme oil^b^, solum oil^c^, plantago bronchial balsam^c^ Diaphragm compress with copper ointment^c^, thyme oil^b^, solum oil^c^
Sleep disorders	Heart compress with aurum-lavandula ointment^c^ Hand or foot bath with lavender bath milk Sternum compress with lavender oil^c^ Embrocation with lavender oil^b^, solum oil^c^, mallow oil^c^ Liver compress with yarrow tea or oil^b^
Exhaustion and weakness	Hand or foot bath with citrus bath milk, rosemary bath milk Liver compress with yarrow tea or oil^b^ Therapeutic wash with citrus bath milk, rosemary bath milk, solum oil^c^ Embrocation with mallow oil^c^, solum oil^c^, rose oil^b^
Depressed mood	Embrocation with mallow oil^c^, rose oil^b^, citrus oil^b^ Therapeutic wash with citrus bath milk Liver compress with yarrow tea or oil^b^
Crisis situation	Hand or foot bath with lavender bath milk Embrocation with rose oil^b^, solum oil^c^, lavender oil^b^ Therapeutic wash with rosemary bath milk
Constipation, meteorism, and irritable bowel syndrome	Abdomen-embrocation with fennel-caraway oil^b^, chamomile oil^b^, melissa oil^c^, oxalis ointment^c^ Abdomen compress with chamomile oil^b^
Edema and congestion	Compress with borago essencec, curd, arnica essence^b^ Embrocation with rosemary oil^b^, solum oil^c^ Therapeutic wash with citrus bath milk
Polyneuropathy	Embrocation with aconite oil^c^, solum oil^c^,
Pain	Embrocation with aconite oil^c^, solum oil^c^, arnica essence^c^ Warm or cool compress
Nausea and vomiting	Abdomen compress with fennel-caraway oil^b^, chamomile oil^b^, melissa oil^c^ Embrocation with fennel-caraway oil^b^, chamomile oil^b^, melissa oil^c^
Restlessness, anxiety, and tension	Heart compress with aurum-lavandula ointment^c^ Liver compress with yarrow tea or oil^b^ Wrist compress with citrus oil^b^ Embrocation with solum oil^c^, lavender oil^b^, rose oil^b^, mallow oil^c^ Sternum compress with lavender oil^b^, arnica essence^c^ Hand or foot bath with lavender bath milk

^a^Selected according to the patient’s symptom burden and preferences.

^b^Essential oils; for embrocations used with a high-quality carrier oil.

^c^Ready-to-use products as anthroposophical products: aconite oil (*Aconitum napellus e tubere ferm 33c Dil. D9 oleos., D-Campher, Lavandulae aetheroleum, Quarz Dil. D9 oleos.*); arnica essence (*Arnica, montana*); aurum-lavandula ointment (*Aurum metallicum praeparatum Dil. D4, Lavandulae aetheroleum, Aetheroleum extractum e floribus recentibus Rosae damascenae et centifoliae*); borago essence (*Borago officinalis ex herba LA 20%*); copper ointment (*Cuprum oxydulatum rubrum*); mallow oil (*Geranii aetheroleum, Malva arborea e floribus W 5%, Hypericum perforatum, Herba rec., Prunus spinosa e floribus W 5%, Sambucus nigra ex umbella W 5%, Tilia platyphyllos/cordata e floribus W 5%*); melissa oil (*Carvi aetheroleum, Foeniculi amari fructus aetheroleum, Melissa officinalis ex herba W 5%, Origanum majorana ex herba W 5%*); oxalis ointment (*Oxalis, folium*); plantago bronchial balsam (*D-Champher, Cera flava, Drosera e planta tota ferm 33c Dil. D3, Eucalypti aetheroleum, Petasites hybridus e radice ferm 33c Dil. D1, Plantago lanceolate e foliis ferm 34 c Dil. D1, Terebinthina laricina, Thymi aetheroleum*); solum oil (*Aesculus hippocastanum e semine LA 25% sicc., Equisetum arvense ex herba LA 20%, Lavandulae aetheroleum, Solum uliginosum*).

### Study Design

A mixed methods research design is used, applying a convergent parallel mixed methods design [52,53]. The decision to use a mixed methods approach was made to enable triangulation of quantitative and qualitative data and to integrate diverse perspectives on the implementation of IN, thereby providing a more comprehensive understanding of the phenomenon. In the field of nursing, mixed methods are also essential for addressing implementation research questions, in addition to single-method approaches [54]. In this convergent parallel design, both types of data are collected simultaneously and with equal priority. They are then evaluated independently and subsequently compared, related, and merged after analysis. This process enables the identification of areas where results converge or diverge, which can be then be explored further during the interpretation phase. The protocol follows the principles outlined in the Good Reporting of a Mixed Methods Study guidelines [55].

This study applies the Consolidated Framework for Implementation Research (CFIR) as its theoretical framework [56,57]. CFIR is a comprehensive framework for studying the implementation of complex interventions in health care settings [58]. The updated version of CFIR includes 5 core domains for analyzing the implementation process [57]: innovation, outer setting, inner setting, individuals, and implementation process. These domains are interrelated and collectively influence the success and sustainability of implementation efforts [56]. However, relying solely on CFIR domains may overlook important contextual influences. Maintaining an open and flexible approach during the implementation process is therefore essential to capture additional relevant factors [58].

To address the primary objectives of the study and reflect the multilevel evaluation approach, 5 interrelated substudies will be conducted, incorporating 3 perspectives: those of patients, relatives, and health care professionals. An overview of these substudies is presented in Table 2, with further details provided in the following sections.

**Table 2:** Study design of the mixed methods approach, comprising 5 interrelated substudies.

Substudy	Aim	Method	Participants
1	To identify perceptions and impact on patient-reported outcomes	Single-arm pre-post questionnaire	Patients
2	To understand how patients experience the IN	Semistructured interviews	Patients
3	To understand how relatives regard the IN	Cross-sectional survey	Relatives
4	To identify facilitators and barriers to the implementation	Semistructured interviews	Nursing and medical staff
5	To demonstrate the feasibility and implementation	Project-related documentation	All project participants

### Substudy 1: Patients’ Perspective (Quantitative)

The aim is to explore patients’ perceptions and experiences with IN interventions, including short-term changes in vitality, sense of warmth and strength, and feelings of calm. The study also seeks to identify the potential impact of IN during the inpatient stay on patient-reported outcomes, such as well-being and associated symptom burden.

#### Study Design

In this substudy, we will conduct a single-arm, exploratory pre-post study with multiple measurement points.

#### Participants

Patients who receive IN interventions during their hospital stay as part of the IMPLEMENT-UKU project are eligible to participate. Additional inclusion criteria are age 18 years or older, capacity to provide informed consent, and provision of written informed consent. Exclusion criteria include pronounced cognitive deficits, an imminent dying process, and inability to participate in the survey due to limited communication abilities, such as insufficient language proficiency. Informed consent will be obtained with particular attention to the acute health conditions of inpatients. To minimize participant burden, questionnaires will be concise, and data collection will be scheduled flexibly. In addition, the questionnaire may be completed with support from study staff if required. Participants retain the right to withdraw from the study at any time without providing justification.

The study is designed as a total population survey, aiming to include all patients participating in the project. However, because not all patients may be able or willing to participate, the resulting sample will be classified as a nonprobability convenience sample. Reasons for nonparticipation in the study will be systematically documented in a screening log. Due to the exploratory nature of this substudy, no formal sample size calculation was performed. The final sample size will be determined by the study duration and the number of eligible patients within this timeframe. We anticipate an eligible population of approximately 20 patients per month, with a projected recruitment rate of 30% and an expected dropout rate of 20%.

#### Data Collection

During the study period, all patients enrolled in the IMPLEMENT-UKU project will be invited to participate on a voluntary basis. During the initial IN consultation, patients will receive detailed information about the study, and the inclusion and exclusion criteria will be reviewed to assess eligibility. If the patient agrees to participate, data will be collected throughout the hospital stay until follow-up after discharge (Figure 1). Participants will first complete a baseline questionnaire (t0). A brief questionnaire (t1) will be administered after each subsequent IN visit with interventions. Following discharge, participants will be asked to complete a follow-up questionnaire (t2). In parallel with the ongoing IN visits, project-related documentation of all applied IN interventions will be conducted as part of substudy 5.

#### Study Outcomes

To comprehensively assess the potential impact of IN on patients’ overall experience and functioning, the study incorporates a set of patient-centered outcomes that capture both physical and psychological dimensions of health. The selected instruments are suitable for use in an inpatient oncology setting and designed to cover a time frame of up to 2 weeks. Outcome parameters include well-being, life satisfaction, health status, symptoms, psychological burden, and self-efficacy. In addition, previous use of and interest in CIM, as well as patients’ perceptions of and satisfaction with IN and its implementation, will be assessed. These outcomes will be measured at 3 time points, as outlined in Table 3.

**Table 3:** Outcomes and instruments used in the quantitative pre-post study with patients (substudy 1). Self-developed items underwent pretesting using the think-aloud method.

Outcomes	Instrument	Items, n	t0 (baseline)	t1 (after each IN visit)	t2 (follow-up)
Well-being	WHO-5^a^	4	✓		✓
Life satisfaction	L-1^b^	3	✓		✓
Health status	SRH^c^	2	✓		✓
Symptoms	NRS^d^	15	✓		✓
Psychological burden	PHQ-4^e^	4	✓		✓
Self-efficacy	SES6G^f^	6	✓		✓
Previous use of CIM^g^	Self-developed	7	✓		
Interest in using CIM	Self-developed	7	✓		
Perception of vitality, warmth, strengthening, coming to rest	NRS	4		✓	
Satisfaction with IN^h^ and implementation of IN	Self-developed	6			✓
Further comments	Open-ended question	1	✓	✓	✓
Sociodemographic data	Demographic data form	6	✓		

^a^WHO-5: WHO-Five Wellbeing Index.

^b^L-1: Short Scale Life Satisfaction.

^c^SRH: self-rated health.

^d^NRS: numeric rating scale.

^e^PHQ-4: Patient Health Questionnaire-4.

^f^SES6G: Self-Efficacy for Managing Chronic Diseases 6-Item Scale.

^g^CIM: complementary and integrative medicine.

^h^IN: integrative nursing.

#### Data Analysis

Missing data will be assessed in terms of extent and patterns. Depending on the amount and nature of missing values, either listwise or pairwise deletion will be used for analysis. In cases of substantial missingness, appropriate imputation methods (eg, multiple imputation) will be considered. All procedures for handling missing data will be reported transparently. Descriptive statistics, including frequency, mean, SD, median, and mode, will be used to characterize the sample and outcome variables. To estimate differences between baseline and follow-up scores, a paired *t* test will be applied; the Wilcoxon signed-rank test will be used as a nonparametric alternative. Additionally, subgroup analyses will be conducted based on variables such as gender, age group, diagnosis, previous experience with CIM, and type of IN intervention. Statistical significance will be defined as *P*<.05.

### Substudy 2: Patients’ Perspective (Qualitative)

This substudy aims to explore how patients perceive and experience IN interventions, as well as their level of satisfaction with the implementation of IN. The primary focus is an in-depth examination of individual perceptions to gain a more nuanced understanding of how IN interventions are received and experienced from the patient’s perspective.

#### Study Design

In this substudy, we will use a qualitative approach, and semistructured interviews will be conducted.

#### Participants

Patients who received IN interventions during their inpatient stay will be invited to participate in interviews after discharge. Additional inclusion criteria are age 18 years or older, capacity to provide informed consent, and provision of written informed consent. Exclusion criteria include pronounced cognitive deficits, an imminent dying process, and limited ability to communicate (eg, insufficient language proficiency).

Purposive sampling will be used to identify and select cases that are rich in information [59]. Participants will be selected to ensure variation in factors such as sex, age, and number of IN interventions received, thereby capturing a wide range of perspectives on the implementation of IN. A minimum of 12 interviews is initially estimated to capture sufficient diversity of patient experiences to address the research questions [60]. However, sampling will be flexible and will continue until thematic saturation is reached, defined as the point at which no new significant themes, concepts, or insights emerge from the data.

#### Data Collection

During their hospital stay, patients will be informed verbally and in writing by study staff. After discharge, they will be contacted by telephone to confirm their interest in participating and to clarify their preferred format and scope of the interview. Upon providing written informed consent, participants will be interviewed online, by telephone, or in person, according to their preference. A semistructured interview format guided by an interview guideline will be used. Each interview will last approximately 45 minutes and will be audio recorded. In addition, the interviewer will take structured notes using a standardized interview protocol to capture relevant contextual details for later analysis. To support sample characterization, the following variables will be recorded: age, sex, marital status, number of children, occupation, and number and type of IN interventions received.

#### Interview Guideline

The interview guideline was developed based on research interests, following the principle of “collecting, checking, sorting, subsuming” [61], and with consideration of CFIR [57]. The guideline will address the following four main topics: (1) previous experience with and use of CIM and IN, (2) perception and experience with IN at both the physical and psychological levels, (3) satisfaction with the implementation of IN during the hospital stay, and (4) perspectives on the future use of IN, including potential integration into everyday life. At the end of the interview, participants will also be invited to provide additional comments or reflections.

#### Data Analysis

Audio recordings will be transcribed according to the simple transcription guidelines proposed by Dresing and Pehl [62]. Any personally identifiable information will be anonymized to ensure data protection. Data will be analyzed using structuring qualitative content analysis as proposed by Kuckartz [63], which combines deductive and inductive category development. Deductive categories will be derived from the research questions and CFIR, while additional inductive categories will emerge from the data. The coding system will be redefined iteratively throughout the analysis process. To enhance credibility and reliability, at least 2 researchers will be involved in coding and interpretation. Initially, a subset of transcripts will be coded independently, and the resulting codes and themes will be compared and discussed to reach consensus. The remaining data will then be coded through a consensus-driven approach by the research team. Data analysis will be performed using MAXQDA (VERBI Software). Upon completion of the analysis, all audio files will be permanently deleted to ensure compliance with data protection regulations.

### Substudy 3: Relatives’ Perspective

Relatives are considered key stakeholders in exploring the feasibility and relevance of transferring low-threshold IN interventions into the home care setting after hospital discharge. The aim of this substudy is to examine relatives’ perspectives on the use of, attitudes toward, and interest in CIM, as well as their perceptions of IN for inpatients.

#### Study Design

In this substudy, we will conduct an anonymous, cross-sectional survey using a quantitative descriptive research design.

#### Participants

The survey will include all relatives of patients who receive IN interventions as part of the IMPLEMENT-UKU project at the hospital. Eligibility criteria for participation are age 18 years or older, sufficient language proficiency, being a relative of a patient received IN interventions, and provision of informed consent.

#### Data Collection

Relatives will be invited to participate in the survey through information flyers and direct contact by nurses involved in the project, who will provide verbal and written information about the study. Participation in the survey is possible using a paper-based questionnaire or a web-based version. The online survey is administered using the Unipark platform (Tivian XI GmbH). Participants will access the questionnaire using a survey link or QR code, and responses will be submitted anonymously.

#### Study Outcomes

The questionnaire includes the following domains: sociodemographic data (2 items), caregiving support (3 items), definition of CIM (2 items), previous use of CIM (12 items), interest in using CIM (12 items), perception of IN at the hospital (5 items), involvement of relatives in project implementation (2 items), and space for additional comments (1 item).

#### Data Analysis

Missing data will be assessed in terms of frequency and distribution across all variables. Cases with missing values will be excluded using listwise or pairwise deletion, as appropriate. Descriptive statistical methods will be used to analyze the data. Depending on the level of measurement, absolute and relative frequencies, means, SDs, and medians will be calculated.

### Substudy 4: Medical and Nursing Staffs’ Perspective

The involvement of health care professionals, including nursing and medical staff on the participating wards, is essential for capturing implementation processes and complementing the perspectives of patients who have received IN interventions. The objective of this substudy is to explore the facilitators and barriers to the implementation of IN from the perspective of health care professionals, as well as their perceptions of its effects and its impact on routine clinical practice.

#### Study Design

In this substudy, we will use a qualitative approach, and semistructured interviews will be conducted.

#### Participants

Nursing and medical staff from the wards participating in the IMPLEMENT-UKU project, as well as the integrative nurses delivering the IN interventions, will be invited to participate. Additional eligibility criteria include age 18 years or older, sufficient language proficiency, employed on a participating ward or within the project, and provision of informed consent.

A purposive sampling strategy will be used to identify and select participants who can provide rich and relevant information [59]. The sampling approach will aim for maximum variation in key characteristics such as age, professional background, and years of employment, to capture a broad range of perspectives. This study aims to conduct a minimum of 12 interviews to collect sufficient diverse perspectives to address the research questions [60]. Sampling will continue until thematic saturation is reached, defined as the point at which no new significant themes, concepts, or insights emerge from the data.

#### Data Collection

Potential participants will be contacted through personal communication, mail, or telephone. Ward managers will serve as gatekeepers during the recruitment process. Nurses and physicians will be informed verbally and in writing by the study staff before the interview. Upon providing written informed consent, interviews will be conducted online, by telephone, or in person, according to the participant’s preference. The interviews will be conducted using a semistructured guideline. Each interview will last approximately 45 minutes and will be audio-recorded. Additionally, the interviewer will take structured notes using an interview protocol to capture relevant contextual aspects for later analysis. The sample description will include age, sex, profession, years of professional experience, and additional CIM training.

#### Interview Guideline

The interview guideline was developed based on research interests, following the principle of “collecting, checking, sorting, subsuming” [61] and with consideration of CFIR [57]. The semistructured interview with nursing and medical staff will cover the following key topics: (1) the process of implementing the IN project, (2) perception and experience with IN on the ward, (3) support and enabling factors to IN implementation, (4) impediments and barriers to IN implementation, and (5) suggestions for future improvements. Participants will also be invited to share additional comments and reflections at the end of the interview.

#### Data Analysis

Audio recordings will be transcribed using the simplified transcription guidelines outlined by Dresing and Pehl [62]. Any information that could reveal participants’ identities will be anonymized during this process. Qualitative data will be analyzed using structuring content analysis according to Kuckartz [63], which combines both deductive and inductive category development. Deductive categories will be derived from the study’s research objectives and CFIR, while additional inductive categories will be identified directly from the data. The category system will be refined iteratively during the coding process. A subset of data will be independently coded by at least 2 researchers, after which the code systems and thematic patterns will be compared and discussed. The remaining data will be coded collaboratively using a consensus-driven approach. Analysis will be conducted using MAXQDA (VERBI Software). In accordance with data protection regulations, all audio files will be permanently deleted upon completion of the analysis.

### Substudy 5: Project-Related Data

In addition to primary data sources, routinely collected data from the IMPLEMENT-UKU project will be used to complement the analysis and assess the feasibility and real-world implementation of IN. This substudy focuses on key implementation indicators, including acceptance, study participation rate, intervention fidelity, and the number of IN visits and interventions performed. Furthermore, the analysis will support the identification of required resources and potential cost factors associated with implementation in routine care.

#### Study Design

In this substudy, we will conduct a retrospective analysis using project-related routine data.

#### Participants

The sample will include all requests for IN consultations and all patients who participated in the project during the study period.

#### Data Collection

Project-related routine documentation will serve as the data source for all IN consultation requests, as well as the corresponding IN visits and interventions. Data for participants in substudy 1 will be collected pseudonymously, while data for all other project participants will be extracted anonymously.

#### Study Outcomes

All parameters shown in Textbox 1 will be collected from project-related routine data. This includes sociodemographic and clinical data, details on IN consultation requests and rejections, records of IN visits and interventions performed, and study participation. These data will be used to analyze the acceptance rate of the IN offer, the study inclusion rate, and intervention fidelity, defined as the extent to which the interventions are delivered in accordance with the standard operating procedure (eg, duration), and to estimate the resources and costs associated with implementing IN.

**Collection of project-related routine data (substudy 5).**

**Sociodemographic and clinical data**

- Sex, age group, ward, and diagnosis group (*International Statistical Classification of Diseases and Related Health Problems, Tenth Revision* [*ICD-10*])

**Integrative nursing (IN) consultation service**

- Reason for IN consultation request, acceptance or rejection of the IN offer, and reasons for rejection

**IN visits**

- Number of visits per patient, duration, notes of preliminary conversation, patient symptoms perceived or reported

**IN interventions**

- Number of interventions per patient, duration, location (body region), type, substances used, perceived or reported reactions after the IN intervention, and side effects

**Study participation**

- Study inclusion and reasons for noninclusion of patients

#### Data Analysis

Where feasible, complete case analysis will be performed; otherwise, pairwise deletion will be applied. Quantitative data will be analyzed using descriptive statistics, including frequencies, medians, means, and SDs, as appropriate. Subgroup analyses (eg, by sex, age group, diagnosis, or type of IN intervention) will be exploratory in nature. Group comparisons will be conducted using independent samples *t* tests for interval-scaled variables and chi-square tests for categorical variables. If the assumptions for parametric tests are not met, nonparametric alternatives such as the Mann-Whitney *U* test and Fisher exact test will be used. The significance level will be set at 5% (*P*<.05). Qualitative data from the open-ended text fields will be categorized using thematic content analysis.

### Mixed Methods Analysis

After the separate analysis of each sub-study, the results will be integrated to generate meta-inferences. The ensuing discussion will examine areas of convergence, divergence, complementarity, and the relationships between the quantitative and qualitative findings, as well as different perspectives on the implementation of IN. The interpretation will be grounded in comparisons and correlations [52,64].

Triangulation approaches will be used to compare and integrate data, enabling both cross-validation and enrichment of the findings [65]. Methodological triangulation will enhance the credibility of results by identifying consistencies and discrepancies across different types of data, such as comparing quantitative and qualitative findings on patients’ perspectives regarding the implementation of IN. Data source triangulation will be applied by incorporating insights from patients, relatives, and health care professionals, enabling a more comprehensive understanding of the implementation of IN for inpatients.

Integrative data analysis will use joint displays to present and synthesize findings, enabling further conclusions [66,67]. A joint display is a matrix that visually aligns and compares results from different data types to support systematic integration. In this study, which follows a convergent design, a side-by-side comparison will be used [67]. Joint displays will be thematically structured, such as according to the domains of to CFIR [57]. These displays will support the development of new insights and conclusions that extend beyond the individual substudies, serving as a foundation for interpretation, discussion, and stakeholder dialogue.

During the integration process, discrepancies between qualitative and quantitative findings will be systematically examined. Rather than being regarded as limitations, these divergences will be considered opportunities for deeper interpretation. They will be mapped in joint displays and explored through iterative team discussions to generate plausible explanations, including contextual factors or subgroup effects. This process will be transparently documented to ensure rigor.

### Ethical Considerations

The evaluation study was conducted in accordance with the Declaration of Helsinki and received ethical approval from the Ethics Committee of the University of Ulm, Germany (144/23). All participants will receive detailed verbal and written information about the project and study objectives as part of the informed consent process. Informed consent will be obtained before participation, ensuring that participation is entirely voluntary. Participants may withdraw from the study at any time without providing a reason and without facing any consequences.

## Results

Initially, the results of each substudy will be analyzed independently. Thereafter, the findings will be integrated and triangulated across the substudies, incorporating both quantitative and qualitative data, as well as the perspectives from different stakeholders, to develop comprehensive meta-inferences. The recruitment phase lasted from October 2023 to January 2025. Data collection was completed in March 2025. As of October 2025, after data verification and plausibility checks, data analysis is ongoing. The first results are expected to be published in 2026.

## Discussion

### Overview

This study outlines the protocol for mixed methods research aimed at making a theory-based contribution to the implementation of IN for inpatients and examining its potential effects at the patient level. The following anticipated findings are derived from the study’s research questions: IN visits and interventions are expected to be implemented as intended, with a moderate to high number of IN consultation requests and repeated IN visits and interventions per patient during the hospital stay. Acceptance rates among patients and staff are likely to be high, with health care professionals perceiving IN as a meaningful complement to conventional care. Contextual, structural, and organizational factors are anticipated to act as key barriers and facilitators in the implementation process. Initial results are expected to show improvements in well-being, reductions in symptom burden, and increased self-efficacy. IN interventions are anticipated to be perceived as supportive and not associated with additional burden. Patients may also report short-term subjective benefits such as enhanced vitality, a sense of warmth and strength, and feelings of calm following IN interventions.

The findings can subsequently be compared and contextualized with studies that have implemented CIM or IN in other settings, such as pediatric care [37,38,40,41,68-70] and outpatient oncology care [35,42,71,72], as well as with studies and programs offering IO for patients [33,73-75]. Situating the findings within the framework of implementation science is essential for advancing both the conceptual development and practical integration of IN into routine care [76]. This research is expected to make a substantial contribution to the implementation of IN as a service for inpatients. Furthermore, the findings will support the German Research Agenda for Nursing Oncology and help establish a scientific foundation for IN in oncology care [43].

The findings of this study will first be shared with the interdisciplinary IMPLEMENT-UKU Steering Committee. The results are expected to be suitable for publication in peer-reviewed journals and for presentation at national and international conferences. As part of the dissemination strategy, the results of the individual substudies will be published separately, followed by an integrative mixed methods publication synthesizing the overall findings. In addition, the results will be shared with the general public through patient forums, information events, and the project website. The findings will also inform the ongoing development and improvement of the IMPLEMENT-UKU project’s implementation strategy. The findings may contribute to the development of practical guidelines to support the standardization and facilitation of IN implementation in other hospital settings. Moreover, the study outcomes are expected to inform future research strategies in the fields of IN and implementation science.

### Limitations

The monocentric design of this study may limit the generalizability of findings to other settings and reduce external validity, as the results reflect the specific implementation context at Ulm University Hospital. Furthermore, due to the patient-centered and multimodal nature of the IMPLEMENT-UKU project, the delivery of IN interventions is not fully standardized and may vary between patients. To ensure consistency, all IN interventions are guided by a symptom-driven IN catalog, which supports targeted and structured application. In addition, a standard operating procedure is in place, and all applied IN interventions are documented in detail through project-specific documentation, enabling analysis of IN intervention variability and its impact.

As participation in this study is voluntary, there is potential for recruitment bias and corresponding selection bias. Although a convenience sampling approach will be used, several strategies will mitigate these limitations. Recruitment and sampling procedures will be transparently documented, and reflexivity will be maintained throughout the recruitment and data analysis phases. Reasons for exclusion and refusal to participate will be systematically recorded. For the qualitative interviews, efforts will be made to ensure heterogeneity in key participant characteristics.

As the studies rely primarily on self-report measures, risk of response bias, recall bias, and social desirability bias must be considered. In substudy 3, the cross-sectional design and convenience sampling of relatives from a single hospital limit external validity and generalizability. Substudy 1 uses a pre-post design without a control group. While this design enables the observation of intra-individual changes over time, it does not permit causal inference, as observed effects cannot be attributed solely to the intervention, thereby limiting internal validity. Accordingly, only exploratory analyses are planned to generate preliminary insights into potential effects and inform future research directions. Nevertheless, triangulation across multiple data sources within the patient group will enhance the credibility and trustworthiness of the findings, despite the nonprobabilistic sampling.

## Abbreviations

CFIR: Consolidated Framework for Implementation Research
CIM: complementary and integrative medicine
IMPLEMENT-UKU: Implementation of Integrative Nursing at the Ulm University Hospital
IN: integrative nursing
IO: integrative oncology
